# Safety reporting quality in multiple sclerosis clinical trials: A review of phase III clinical trials included in FDA approval of disease-modifying treatments

**DOI:** 10.1177/20552173251390649

**Published:** 2025-10-29

**Authors:** William Z. Lin, Jaimie J. Lee, Anibal Chertcoff, Helen Tremlett, John L. K. Kramer

**Affiliations:** Department of Anesthesiology, Pharmacology, and Therapeutics, Faculty of Medicine, 8166 University of British Columbia, Vancouver, BC, Canada; 507272 International Collaboration on Repair Discoveries (ICORD), University of British Columbia, Vancouver, BC, Canada; Department of Pharmacology and Toxicology, Temerty Faculty of Medicine, 7938 University of Toronto, Toronto, ON, Canada; Department of Anesthesiology, Pharmacology, and Therapeutics, Faculty of Medicine, 8166 University of British Columbia, Vancouver, BC, Canada; 507272 International Collaboration on Repair Discoveries (ICORD), University of British Columbia, Vancouver, BC, Canada; Division of Neurology, Faculty of Medicine, 12358 University of British Columbia, Vancouver, BC, Canada; 215463 Djavad Mowafaghian Centre for Brain Health, University of British Columbia, Vancouver, BC, Canada; Department of Internal Medicine, Health Sciences Centre, 12359 Max Rady College of Medicine, University of Manitoba, Winnipeg, MB, Canada; Division of Neurology, Faculty of Medicine, 12358 University of British Columbia, Vancouver, BC, Canada; 215463 Djavad Mowafaghian Centre for Brain Health, University of British Columbia, Vancouver, BC, Canada; Department of Anesthesiology, Pharmacology, and Therapeutics, Faculty of Medicine, 8166 University of British Columbia, Vancouver, BC, Canada; 507272 International Collaboration on Repair Discoveries (ICORD), University of British Columbia, Vancouver, BC, Canada; 215463 Djavad Mowafaghian Centre for Brain Health, University of British Columbia, Vancouver, BC, Canada

**Keywords:** Safety reporting, adverse events, clinical trials, multiple sclerosis, disease-modifying therapies

## Abstract

**Background:**

Evaluating safety of emerging interventions is important in clinical trials. To support reporting of safety outcomes, a harms extension of the Consolidated Standards of Reporting Trial (CONSORT) guidelines was published in 2004.

**Objective:**

To examine safety reporting in pivotal trials of disease-modifying therapies (DMTs) in patients with multiple sclerosis (MS).

**Methods:**

Published phase III clinical trials from 1995 to 2022 included in FDA approval material for MS DMTs were compiled and reviewed by two independent examiners. Criteria derived from the CONSORT harms extension were used to evaluate safety reporting. Linear regression was applied to examine associations between quality of safety reporting and study level factors.

**Results:**

30 publications were included in the analysis. Overall, safety reporting quality was fair with an average score of 10.2 out of 15. Trials examining small molecule versus biologic interventions (*p* = 0.001) and recent publication (*p* = 0.005) were associated with higher quality reporting. Items related to laboratory-defined toxicity and defining adverse events were among reporting items notably lacking (present in 53% and 40% of publications, respectively).

**Conclusion:**

While the reporting of phase III clinical trials for DMTs for the treatment of MS has improved with time, there remain gaps and opportunities for further improvement.

## Introduction

The basis of drug approval is, in part, related to the conduct of pivotal phase III clinical trials.^1^ Principally designed to test efficacy, phase III clinical trials also provide important insights into safety. In parallel to the submission for drug approval, manuscripts outlining efficacy and safety results are often submitted for peer review. These play important roles in synthesizing primary and secondary outcomes of the trial to the wider scientific community and serve as source material for meta-analyses. To support quality reporting of clinical trials, seminal guidelines were published in 1996, with the most recent revision published in 2010.^2,3^ While improving the overall quality of reporting,^4^ concerns were subsequently raised with regard to safety. The lack of attention paid to safety was highlighted in one noteworthy study, which observed that space dedicated to reporting harms in phase III clinical trial publications was similar to that occupied by author affiliations.^5^ In light of major deficiencies, a “harms” extension was added to original clinical trial reporting guidelines.^6^ While regulatory agencies consider the entirety of safety data provided by trials, the purpose of adequate safety reporting in manuscripts is not necessarily about the safety of the medication in question; rather, quality communication of safety allows researchers and clinicians to critically appraise the trial results and enables them to make informed decisions.^6^

The past 30 years have seen a number of disease-modifying therapies (DMTs) for multiple sclerosis (MS) approved by regulatory agencies in North America and Europe. These typically are either immunomodulators or immunosuppressants, whose anti-inflammatory properties decrease MS relapse frequency and slow disability.^1^ Accompanying benefits of intervention is the possibility for serious adverse reactions. Chief among concerns include but are not limited to progressive multifocal leukoencephalopathy, cardiac arrhythmias, hepatotoxicity, secondary autoimmunity, and malignancies.^7^ There are potential disparities in how these adverse effects are reported from publication to publication, and to our knowledge, the quality of reporting in the field has not been examined.

The objective of the current study was to evaluate the quality of safety reporting in phase III clinical trials contributing to the FDA approval of a DMT for the treatment of MS. After identifying relevant trials, safety reporting was reviewed according to established guidelines. A secondary analysis included examining study level factors associated with reporting quality. We hypothesized that year of publication would be the major factor associated with safety reporting quality.

## Material and methods

### Identification of disease-modifying therapies

Twenty DMTs that had ever been approved by the FDA for the treatment of MS were identified as of 1 June 2023. These DMTs were compiled by cross-referencing key published articles with the FDALabel database (Table 1).^1,8,9^ The type of drug (biologic or small molecule) was determined by referencing the FDA approval package for the drug. These drug approval packages contain information on all trials that were used to inform the regulatory review and approval of new drugs.^10^ Biologic drugs and small molecule drugs were classified according to their FDA approval process (Biologics License Application and New Drug Application, respectively). This distinction of small molecule versus biologic drugs was made, in part to allow for comparison with other studies that made the same distinction.

**Table 1.** List of disease-modifying therapies that had ever been approved by the FDA for the treatment of multiple sclerosis through until 1 June 2023 (including daclizumab, withdrawn from market in 2018).

Generic name	Brand name	Type of drug	Indication (FDA)	Active ingredient previously approved for other indication (FDA)
Alemtuzumab	Lemtrada	Biologic	RRMS, SPMS	Yes
Cladribine	Mavenclad	Small molecule	RRMS, SPMS	Yes
Daclizumab	Zinbryta	Biologic	RRMS	Yes
Dimethyl fumarate	Tecfidera	Small molecule	CIS, RRMS, SPMS	No
Diroximel fumarate	Vumerity	Small molecule	CIS, RRMS, SPMS	No
Fingolimod	Gilenya	Small molecule	CIS, RRMS, SPMS (ages 10 years and older)	No
Glatiramer acetate	Copaxone	Small molecule	CIS, RRMS, SPMS	No
Interferon beta-1a	Rebif, Avonex	Biologic	CIS, RRMS, SPMS	No
Interferon beta-1b	Extavia, Betaseron	Biologic	CIS, RRMS, SPMS	No
Mitoxantrone	Novantrone	Small molecule	RRMS, SPMS, PRMS	Yes
Monomethyl fumarate	Bafiertam	Small molecule	CIS, RRMS, SPMS	No
Natalizumab	Tysabri	Biologic	CIS, RRMS, SPMS	No
Ocrelizumab	Ocrevus	Biologic	CIS, RRMS, SPMS, PPMS	No
Ofatumumab	Kesimpta	Biologic	CIS, RRMS, SPMS	Yes
Ozanimod	Zeposia	Small molecule	CIS, RRMS, SPMS	No
Peginterferon beta-1a	Plegridy	Biologic	CIS, RRMS, SPMS	No
Ponesimod	Ponvory	Small molecule	CIS, RRMS, SPMS	No
Siponimod	Mayzent	Small molecule	CIS, RRMS, SPMS	No
Teriflunomide	Aubagio	Small molecule	CIS, RRMS, SPMS	No
Ublituximab	Briumvi	Biologic	CIS, RRMS, SPMS	No

*CIS*: clinically isolated syndrome; *RRMS*: relapsing-remitting multiple sclerosis; *SPMS*: secondary progressive multiple sclerosis; *PPMS*: primary progressive multiple sclerosis; *PRMS:* progressive-relapsing multiple sclerosis.

After compiling the drugs, the Drugs@FDA database was queried to find the original approval packages. On the basis of this information, pivotal phase III clinical trials included in the FDA approval were identified. A total of 33 trials were identified through this process, whose results were published in 30 articles (Table 2). Trials for indications other than MS were not included, nor were any subsequent trials cited in the product monograph after initial approval (Supplemental Table 1).

**Table 2.** Characteristics of the included randomized-controlled clinical trials of disease-modifying therapies for the treatment of multiple sclerosis.

Trial name(s)	Intervention	Indication	N	Comparator group	Double-blinded?
CARE-MS I	Alemtuzumab	RRMS	563	Interferon beta-1a SC	No
CARE-MS II	Alemtuzumab	RRMS	798	Interferon beta-1a SC	No
CLARITY	Cladribine	RRMS	1326	Placebo	Yes
DECIDE	Daclizumab	RRMS	1841	Interferon beta-1a IM	Yes
DEFINE	Dimethyl fumarate	RRMS	1234	Placebo	Yes
CONFIRM	Dimethyl fumarate	RRMS	1417	Placebo, glatiramer acetate	Yes
EVOLVE-MS I	Diroximel fumarate	RRMS	696	Dimethyl fumarate	No
EVOLVE-MS II	Diroximel fumarate	RRMS	504	Dimethyl fumarate	Yes
FREEDOMS	Fingolimod	RRMS	1272	Placebo	Yes
TRANSFORMS	Fingolimod	RRMS	1292	Interferon beta-1a IM	Yes
CMSSG	Glatiramer acetate	RRMS	251	Placebo	Yes
PRISMS	Interferon beta-1a SC	RRMS	560	Placebo	Yes
EVIDENCE	Interferon beta-1a SC	RRMS	677	Interferon beta-1a IM	No
MSCRG	Interferon beta-1a IM	RRMS	301	Placebo	Yes
CHAMPS	Interferon beta-1a IM	CIS	383	Placebo	Yes
MSSG	Interferon beta-1b SC	RRMS	372	Placebo	Yes
MIMS	Mitoxantrone	SPMS	194	Placebo	Yes
AFFIRM	Natalizumab	RRMS	942	Placebo	Yes
SENTINEL	Natalizumab	RRMS	1171	Placebo	Yes
OPERA I/OPERA II	Ocrelizumab	RRMS	821/835	Interferon beta-1a SC	Yes
ORATORIO	Ocrelizumab	PPMS	732	Placebo	Yes
ASCLEPIOS I/ASCLEPIOS II	Ofatumumab	RRMS, SPMS	927/955	Teriflunomide	Yes
SUNBEAM	Ozanimod	RRMS, SPMS, PRMS	1346	Interferon beta-1a IM	Yes
RADIANCE	Ozanimod	RRMS, SPMS, PRMS	1313	Interferon beta-1a IM	Yes
ADVANCE	Peginterferon beta-1a	RRMS	1512	Placebo	Yes
OPTIMUM	Ponesimod	RRMS, SPMS	1133	Teriflunomide	Yes
EXPAND	Siponimod	SPMS	1645	Placebo	Yes
TEMSO	Teriflunomide	RRMS, SPMS	1088	Placebo	Yes
TOWER	Teriflunomide	RRMS, SPMS	1169	Placebo	Yes
ULTIMATE I/ULTIMATE II	Ublituximab	RRMS, SPMS	1094	Teriflunomide	Yes

*CIS*: clinically isolated syndrome; *RRMS*: relapsing-remitting multiple sclerosis; *SPMS*: secondary progressive multiple sclerosis; *PPMS*: primary progressive multiple sclerosis; *PRMS:* progressive-relapsing multiple sclerosis*; IM*: intramuscular; *SC*: subcutaneous.

### Data extraction

Data extraction focused on information pertaining to safety information reported in the original clinical trial. To align with our previous work,^11^ previous reporting criteria were adopted and modified from Sivendran et al.,^12^ which was based on the 10-item checklist from the Consolidated Standards of Reporting Trial (CONSORT) statement harms extension (Table 3).^6^ A further item was added following a preliminary review, derived from the original criterion (specifying the instrument or scale used to categorize adverse events (AEs)), which was split into evaluating laboratory toxicities and other AEs separately (items 4 and 5) due to differences in reporting between the two. For item 15, examples of generic or vague descriptors of toxicity included unclear terminology (such as “elevated liver enzymes” without defining what counted as elevation), describing the regimen as “well tolerated,” little discussion of different toxicities encountered, or little to no discussion of toxicity at all. Each article was evaluated with and without taking supplementary information into account, according to the checklist listed in Table 3 for a total score out of 15. A second set of criteria (total score of 18) was adapted from Hadi et al. (2017) for the purpose of ensuring validity and comparing different interpretations of the same criteria.^13^ The quality of safety reporting in each trial was evaluated according to the checklist by two independent reviewers (WZL and JJL). Any discrepancies between the two evaluations were resolved by JLKK.

**Table 3.** List of adverse event reporting criteria (adapted from Sivendran et al., 2014; Ioannidis et al. 2004).

**Paper Section**	**CONSORT Harms extension checklist**	**Criteria List (adapted from Sivendran et al., 2014)**
Title and abstract	1. If the study collected data on harms and benefits, the title or abstract should so state	1. Title or abstract states if adverse events are addressed in the article
Introduction	2. If the trial addresses both harms and benefits, the introduction should so state	2. Introduction or purpose states if adverse events are addressed in the article
Methods	3. List addressed adverse events with definitions for each (with attention, when relevant, to grading, expected vs. unexpected events, reference to standardized and validated definitions, and description of new definitions)	3. Article specifies if the reported adverse events include all recorded events or only a selected sample
		4. Article specifies instrument or scale used to categorize lab-defined toxicities
		5. Article specifies instrument or scale used to categorize all other adverse events
	4. Clarify how harms-related information was collected (mode of data collection, timing, attribution methods, intensity of ascertainment, and harms-related monitoring and stopping rules, if pertinent)	6. Article specifies surveillance time frame for adverse events
		7. Article specifies if early stopping rule was used in study
	5. Describe plans for presenting and analyzing information on harms (including coding, handling of recurrent events, specification of timing issues, handling of continuous measures, and any statistical analyses)	8. Article specifies if recurrent events in the same patient are counted as single or separate events
Results	6. Describe for each arm the participant withdrawals that are due to harms and their experiences with the allocated treatment	9. Article reports reasons for treatment discontinuations
		10. Article reports if deaths related to adverse events have occurred
	7. Provide the denominators for analyses on harms	11. Article specifies which patients were evaluable for toxicity
		12. Article reports absolute numbers of adverse events
	8. Present the absolute risk per arm and per adverse event type, grade, and seriousness, and present appropriate metrics for recurrent events, continuous variables, and scale variables, whenever pertinent	13. Article reports all adverse events and not only those above a threshold
		14. Article does not combine adverse events of varying severity
	9. Describe any subgroup analyses and exploratory analyses for harms	Not included: only valid in publications including subgroup analyses
Discussion	10. Provide a balanced discussion of benefits and harms with emphasis on study limitations, generalizability, and other sources of information on harms	15. Article does not use generic or vague descriptors of harms

Study information was also extracted from each manuscript, including year of publication (as a continuous variable), journal, number of participants, location of trial (international or single country), type of intervention (biologic drug or small molecule drug), funding source (industry, government, or a combination of the two), and the comparator group used in the study (placebo or single-arm trial versus active comparator). The journal impact factor was determined by reviewing Clarivate Journal Citation Reports 2019. If not stated in the original publication, the type of drug and previous approval of the active component for another indication were determined by a review of the Drugs@FDA database. Study level factors associated with safety reporting were examined with multi-variable linear regression. The total scores from the safety reporting review were included as the dependent variable. Paired t-tests were used to compare scores including and excluding supplementary data. Pearson correlations were calculated between scores using criteria adapted from Sivendran et al. (2014) and criteria adapted from Hadi et al. (2017). Statistical significance was reported with two-sided p-values with a significance threshold of 0.05. All statistical tests were performed in R v.4.2.3 and graphs were generated with the ggplot2 package.^14^

## Results

### Study characteristics

Thirty peer-reviewed publications of phase III clinical trial results used in the FDA approval of DMTs for the treatment of MS were reviewed. Fifteen articles used small molecule drugs as the intervention (50%), whereas 15 used biologic drugs (50%) (Table 4). The average number of participants was 1012 (SD = 484, range = 194–1882).

**Table 4.** Study characteristics of included publications (n = 30)

**Characteristic**	**Number of articles (n, %)**
Randomized controlled trial	29 (97%)
Double-blinded	26 (87%)
International	28 (93%)
Industry-sponsored only	27 (90%)
Small molecule drug	15 (50%)
Biologic drug	15 (50%)
Active comparator used	14 (47%)
Active ingredient approved for other indication	6 (20%)
Has supplemental data/published material	23 (77%)

### Safety reporting quality

**Figure 1.**
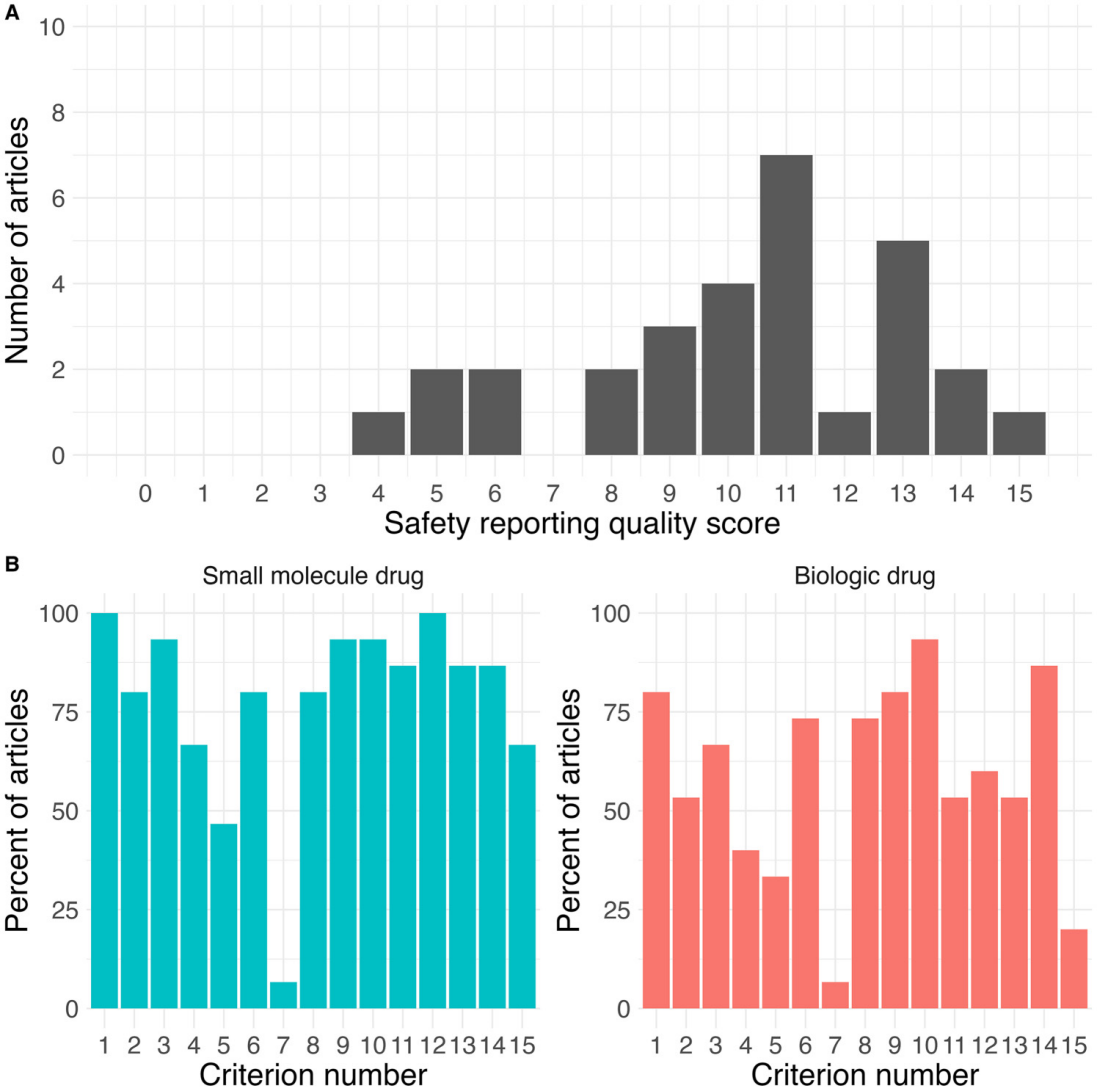
(A) Frequency distribution of safety reporting quality scores (out of 15), not including supplemental data. (B) Percent of articles fulfilling each item in criteria list in Table 3 not including supplemental data, grouped by intervention type (small molecule or biologic drug).

The accordance in scoring between the two reviewers was 98.9% (1424/1440 criteria evaluated). The average safety reporting score for all the manuscripts using the first set of criteria adapted from Sivendran et al. (2014) (out of 15) was 10.2 (SD = 2.9, range = 4–15) (Figure 1). This increased to 10.6 (SD = 3.0, range = 4–15) after taking data from supplemental files into account. The average safety reporting score for manuscripts in secondary analyses using the set of criteria adapted from Hadi et al. 2017 (out of 18) was 13.4 (SD = 3.3, range = 3–18). Scores from each set of criteria were highly correlated (r = 0.816, p < 0.001).

**Figure 2.**
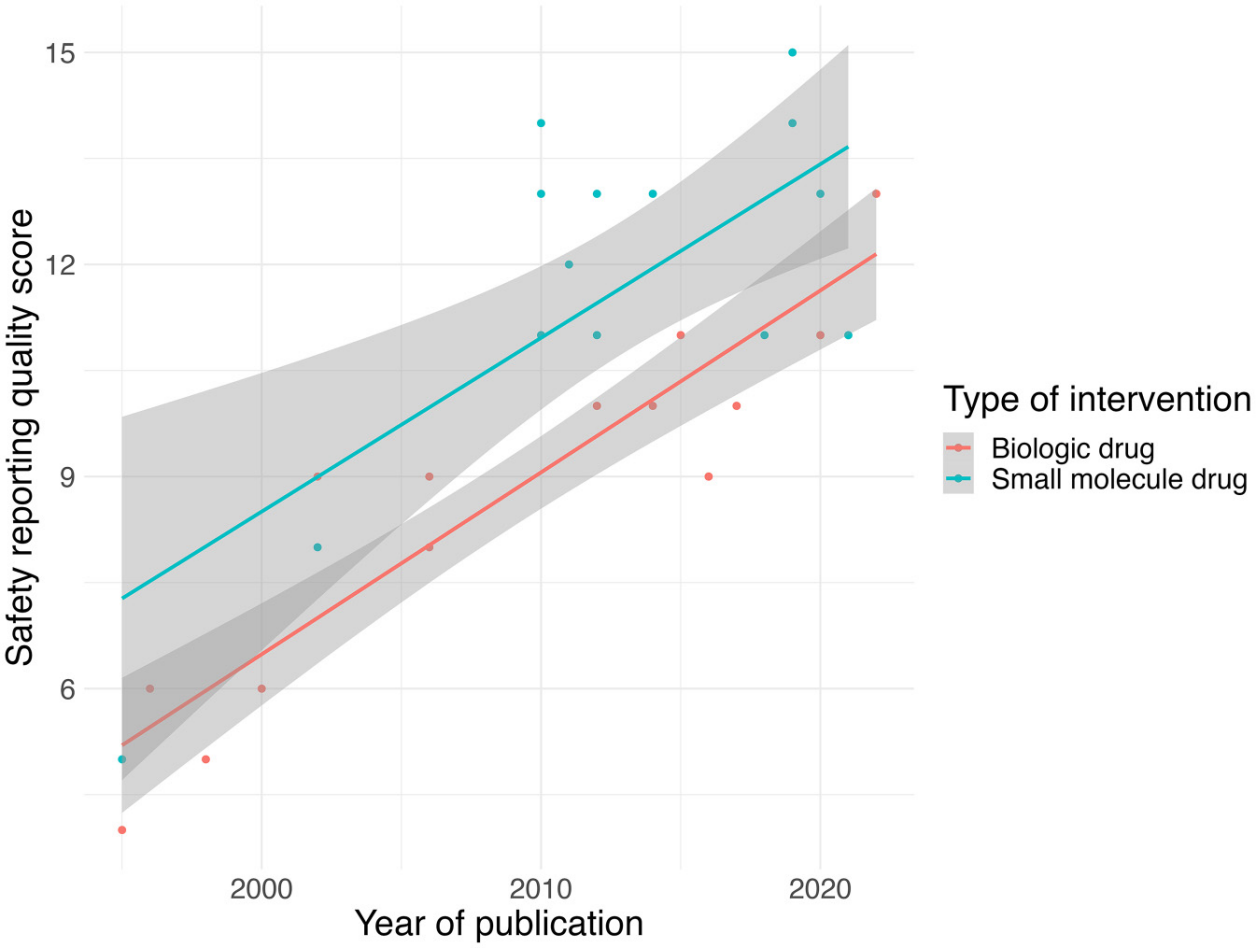
Correlation between safety reporting quality score (from Sivendran et al. 2014) and year of publication, colored by type of intervention.

Analyses on the reporting frequency for each criterion showed that many publications did not specify early stopping rule usage (criteria 7), did not specify the instrument or scale for non-laboratory toxicity AE classification (criteria 5), or included vague descriptors of toxicity (criteria 15), while most publications addressed safety in the abstract or title (criteria 1), reported reasons for treatment discontinuations (criteria 9), and reported any deaths related to AEs (criteria 10) (Figure 1, Table 5). Based on a multi-variable linear regression, the quality of safety reporting was significantly associated with year of publication (p < 0.01) and intervention type, with small molecule drugs having significantly higher scores than biologic drugs (p < 0.01) (Figure 2, Table 6). Journal impact factor, type of trial, double blinding, locations of trial, study funding, type of comparator group, previous approval of active ingredients, and number of participants were not significantly associated with the quality of safety reporting. Taking supplemental data into account resulted in a small but significant increase in mean scores (mean change in score = 0.4, t = 3.062, p = 0.005,) (Supplemental Table 2), but did not affect the overall relationships seen (Supplemental Table 3). Similar results were seen with the second set of criteria; however, intervention type was no longer significant (Supplemental Tables 4 and 5).

**Table 5.** Number of publications (n = 30) fulfilling each criterion in the first set of criteria from Sivendran et al. 2014, grouped by drug type (small molecule, n = 15 and biologics, n = 15), and date of publication (2010 and prior, and after 2010, i.e. year of publication of harms extension).

Criterion	Number of publications fulfilling criterion n (%)						
	Small molecule drugs (n = 15)			Biologic drugs (n = 15)			Total (n = 30)
	2010 and prior (n = 5)	After 2010 (n = 10)	Total (n = 15)	2010 and prior (n = 7)	After 2010 (n = 8)	Total (n = 15)	
1. Title or abstract states if adverse events are addressed	5 (100%)	10 (100%)	15 (100%)	4 (57%)	8 (100%)	12 (80%)	27 (90%)
2. Introduction or purpose states if adverse events are addressed	2 (40%)	10 (100%)	12 (80%)	3 (43%)	5 (63%)	8 (53%)	20 (67%)
3. Article specifies if reported adverse events include all recorded events or selected sample	4 (80%)	10 (100%)	14 (93%)	2 (29%)	8 (100%)	10 (67%)	24 (80%)
4. Article specifies instrument or scale used to categorize lab-defined toxicities	2 (40%)	8 (80%)	10 (67%)	2 (29%)	4 (50%)	6 (40%)	16 (53%)
5. Article specifies instrument or scale used to categorize all other adverse events	1 (20%)	6 (60%)	7 (47%)	0 (0%)	5 (63%)	5 (33%)	12 (40%)
6. Article specifies surveillance time frame for adverse events	5 (100%)	7 (70%)	12 (80%)	6 (86%)	5 (63%)	11 (73%)	23 (77%)
7. Article specifies if early stopping rule was used	0 (0%)	1 (10%)	1 (7%)	1 (14%)	0 (0%)	1 (7%)	2 (7%)
8. Article specifies if recurrent events in the same patient are counted as single or separate events	4 (80%)	8 (80%)	12 (80%)	4 (57%)	7 (88%)	11 (73%)	23 (77%)
9. Article reports reasons for treatment discontinuations	5 (100%)	9 (90%)	14 (93%)	7 (100%)	5 (63%)	12 (80%)	26 (87%)
10. Article reports if deaths related to adverse events have occurred	4 (80%)	10 (100%)	14 (93%)	6 (86%)	8 (100%)	14 (93%)	28 (93%)
11. Article specifies which patients were evaluable for toxicity	5 (100%)	8 (80%)	13 (87%)	4 (57%)	4 (50%)	8 (53%)	21 (70%)
12. Article reports absolute numbers of adverse events	5 (100%)	10 (100%)	15 (100%)	1 (14%)	8 (100%)	9 (60%)	24 (80%)
13. Article reports all adverse events and not only those above a threshold	3 (60%)	10 (100%)	13 (87%)	1 (14%)	7 (88%)	8 (53%)	21 (70%)
14. Article does not combine adverse events of varying severity	3 (60%)	10 (100%)	13 (87%)	5 (71%)	8 (100%)	13 (87%)	26 (87%)
15. Article does not use vague descriptors of toxicity	3 (60%)	7 (70%)	10 (67%)	1 (14%)	2 (25%)	3 (20%)	13 (43%)

**Table 6.** Coefficients table from multiple linear regression of independent variables on safety reporting scores from Sivendran et al. 2014. *p < 0.05, **p < 0.01.

Variable	β [95% confidence interval]	*t*-value	*P*-value
Year of publication	0.528 [0.184, 0.872]	3.209	0.005 **
Journal impact factor	0.118 [−0.161, 0.397]	0.885	0.387
Randomized-controlled trial	0.042 [−0.248, 0.331]	0.301	0.766
Double-blinded	−0.190 [−0.522, 0.142]	−1.200	0.245
Location (single country)	0.064 [−0.344, 0.471]	0.326	0.748
Funding (industry and government)	−0.116 [−0.590, 0.359]	−0.510	0.616
Type of intervention (small molecule drug)	0.451 [0.207, 0.694]	3.875	0.001 **
Comparator group (placebo or *single-arm)	−0.012 [−0.287, 0.264]	−0.087	0.931
Previous approval of active ingredient for other indication	0.030 [−0.256, 0.316]	0.222	0.827
Number of participants	0.184 [−0.169, 0.537]	1.090	0.289

*One single-arm study^17^ was grouped with studies that used placebo for the purposes of the analysis.

## Discussion

Across 30 phase III clinical trial publications for DMTs for the treatment of MS, the quality of safety reporting was generally fair; the overall mean safety reporting quality score was 10.5 out of 15. Reporting of safety was better for small molecule drugs compared to biologics, although improved over time for both. Notable deficiencies in safety reporting included the description of instruments used to categorize laboratory-defined toxicities and AEs (items 4 and 5). Biologics were also deficient, before and after 2010, and often used vague terminology (criterion 15) to characterize safety (e.g. "elevated liver enzymes" without definition). Collectively, our findings highlight elements of safety reporting that are currently done well by authors of the published phase III RCTs of the DMTs for the treatment of MS, as well as key areas for improvement.

The quality of reporting harms in published clinical trials has been examined by others across several different health conditions.^5,12,13,11^ The prevailing outcome of these studies is that despite repeated calls for better adherence to guidelines and indications of improvements over time, there remains potential for better safety reporting in clinical trials. We typically observed similar outcomes for the MS DMT clinical trials, with total scores improving over time. However, major gaps were still present 10 years after the harms guidelines were first introduced. Concerns for reporting harms were particularly evident for the biologic interventions for MS, the reasons for which are unclear. It is possible that given the concerns about long-term safety with biologic medications and their potential for immunogenicity,^15–17^ authors may instead opt to dedicate entire manuscripts to discussing medication safety.

A number of factors need to be considered in view of these findings. The first is that a “lack of awareness” for guidelines, as was initially proposed to explain low adherence, does not likely continue to explain deficiencies. However, the gradual acceptance and endorsement of the CONSORT statement by different journals may have contributed to increasing scores over time. With the recent update of the CONSORT harms statement in 2022 and the incorporation of items from the 2004 statement into the items of the main CONSORT statement,^18^ there may be more awareness and clarity regarding safety reporting in clinical trials in the future.

Previous reviews of clinical trials applying similar criteria for reporting provide an opportunity for comparison with MS.^13,12^ One common finding was that safety reporting quality improved over time. This likely relates to a general increase in awareness among clinical trial investigators regarding safety reporting and widespread adoption of the CONSORT statement by journals. A head-to-head comparison of results indicates a number of areas where published MS DMT clinical trials exceeded those reported for oncology. For example, reporting all AEs was achieved in 72% of the MS DMT clinical trials compared to only 4% in Sivendran et al. 2014. Other scoring criteria, such as avoiding use of vague terminology, were more often fulfilled in oncology publications (65% versus 41% among the MS DMT clinical trials). A review of clinical trials for disease-modifying antirheumatic between 1999 and 2005 likewise found deficiencies in AE definitions, although no significant differences between the small molecule and biologic DMARDs were found when using these criteria.^13^ Specific criteria, such as specifying how harms data was collected, were superior in the MS DMT clinical trials (60%) compared to in rheumatoid arthritis DMARD clinical trials (29%). The differences in scores between the two sets of criteria adapted from different publications suggest differing interpretations of the CONSORT guidelines, which could be remedied with more specific wording in guidelines. In general, our current study combined with others suggests that there were deficiencies in safety reporting across different specialties, although the reporting in phase III clinical trials for the MS DMTs was generally of higher quality.

Another important factor to consider is the emergence of supplemental data files and separate manuscripts pertaining to safety. Space restrictions have long plagued the reporting of harms but are no longer a substantial barrier given the emergence of online and supplemental material, including complete protocols. For a number of interventions, secondary manuscripts have also been published outlining the safety of an intervention. The use of supplemental figures and data can be controversial, as there have been sentiments that any important data (such as safety data in this case) should be reported in the main article and not in the supplemental data files, given that it is not accessed as frequently as the main article.^19^ However, this could arguably also provide a more thorough description of the safety of the intervention that is not restricted by word and figure count limitations in the main article. In our review, we identified a number of studies that only specified the surveillance timeframe or reasons for treatment discontinuation in supplemental files.^20,21^ The inclusion of supplemental data generally increased the scores of papers published after 2010, suggesting that reporting additional safety data in supplemental data files is a relatively recent trend. However, there is little to no research on safety reporting quality taking supplemental data into account, preventing any comparisons with other studies.

Similar to that reported in oncology trials, a few MS DMT clinical trials we reviewed (only 2 of 30) included information on early stopping rules. Defining early stopping rules is, without question, a critical element of planning and designing a clinical trial and protecting the safety of trial participants.^22^ They are often predicated on AEs, laboratory-defined toxicities, or results from interim efficacy analyses. The absence of this information from published studies may have been due to the assumption that these rules are implemented in most clinical trials. Within this study, one article discussed a monitoring committee that had the ability to stop the trial due to safety concerns, while another article discussed an early trial end due to efficacy.^23,24^

Defining laboratory toxicities and other AEs is an area for improvement in reporting safety in future MS trial publications. Meta-analyses of the MS DMT Phase III clinical trials across both small molecule and biologic drugs have noted issues with published data such as inconsistent terminology and the lack of details regarding the definition and severity of AEs, similar to what was found in the present study.^25–27^ This has the effect of decreasing the quality of data available on which to perform safety analyses, ultimately affecting patient recommendations. Concerns regarding reporting these items are not limited to publications, having also been raised in drug approval packages. For example, the clinical reviews included in the approval package for cladribine, a small molecule drug whose phase III results were published in 2010, state that “in many cases, information needed to fully evaluate adverse events was not available.”^27^ Furthermore, there were disagreements regarding AE coding and reasons for treatment discontinuation. The medical reviews for the teriflunomide approval package likewise stated that “there is little description of [adverse events] ” in patient narratives included for submission showing that issues with safety reporting can be present throughout the entire lifespan of a drug.^28^

Limitations of our study included the lack of generalizability to other studies of MS DMTs, as the Phase III trials included in this study were pivotal and had high prominence and importance. Furthermore, some recommendations and items on the checklist may be considered more relevant than others and their inclusion within an article may be constrained due to word limits. In the current analysis, elements with different importance or relevance (e.g. reporting all AEs or reporting number of deaths) were all weighed equally in the checklist (Table 3). To address this, certain elements that may be considered more important or relevant could be weighed more heavily. Additionally, our study only examined data present in the main text and not separate safety publications. Some papers reported the instruments and scales used to classify and grade AEs, the reasons for patient discontinuation, or the complete breakdown of AEs only in the supplemental appendix, and other trials such as that of daclizumab and diroximel fumarate published safety analyses focused exclusively on cutaneous and gastrointestinal AEs, respectively.^29,30^ Dedicated safety publications such as these could arguably increase the reporting quality, especially regarding particular AEs of interest, and not incorporating these and supplemental material in the present analyses may have contributed to the heterogeneity in the results, as well as providing an underestimate of the true safety reporting quality.

The consequences of unsatisfactory safety reporting may introduce difficulties for clinicians and researchers to interpret the data from publications given how AE data are reported. This is relevant particularly in pharmacovigilance, where the reporting of AEs in publications is a crucial element in assessing drug safety before the drug is marketed.^31^ To remedy this, the reporting of AEs may be partially improved by including the CONSORT harms extensions recommendations in the core CONSORT checklist.^32^ However, even with these recommendations, there may still be differences in how AEs are classified and reported between different trials. In the context of MS, it may be possible to adopt a standardized safety reporting protocol tailored for MS to be used in future clinical trials, which would facilitate pharmacovigilance and allow for simplified and less ambiguous comparisons of safety between drugs. The implementation of this would require careful consultation with patients, physicians, and researchers.

## Conclusions

Overall, the quality of safety reporting in phase III MS DMT trials was variable when evaluated against the suggestions of the CONSORT harms extension. The general quality of reporting increased with time and was associated with the type of intervention in question. There is considerable importance in detailed and complete safety reporting as this information is necessary for physicians to make informed decisions about medication choices for patients, and for researchers to thoroughly analyze the safety of different interventions in meta-analyses. This may be accomplished through the development of a standardized reporting procedure for AEs in MS, which could further improve the quality of safety reporting and ease of research in the future.

## Supplemental Material

sj-docx-1-mso-10.1177_20552173251390649 - Supplemental material for Safety reporting quality in multiple sclerosis clinical trials: A review of phase III clinical trials included in FDA approval of disease-modifying treatments Supplemental material, sj-docx-1-mso-10.1177_20552173251390649 for Safety reporting quality in multiple sclerosis clinical trials: A review of phase III clinical trials included in FDA approval of disease-modifying treatments by William Z. Lin, Jaimie J. Lee, Anibal Chertcoff, Helen Tremlett and John L. K. Kramer in Multiple Sclerosis Journal – Experimental, Translational and Clinical

sj-csv-2-mso-10.1177_20552173251390649 - Supplemental material for Safety reporting quality in multiple sclerosis clinical trials: A review of phase III clinical trials included in FDA approval of disease-modifying treatments Supplemental material, sj-csv-2-mso-10.1177_20552173251390649 for Safety reporting quality in multiple sclerosis clinical trials: A review of phase III clinical trials included in FDA approval of disease-modifying treatments by William Z. Lin, Jaimie J. Lee, Anibal Chertcoff, Helen Tremlett and John L. K. Kramer in Multiple Sclerosis Journal – Experimental, Translational and Clinical
