# Supplementary material for: Safety reporting quality in multiple sclerosis clinical trials: A review of phase III clinical trials included in FDA approval of disease-modifying treatments
Source: Mult Scler J Exp Transl Clin. 2025 Oct 29;11(4):20552173251390649. doi: 10.1177/20552173251390649 (PMC12576269; doi:10.1177/20552173251390649)
Supplement: sj-docx-1-mso-10.1177_20552173251390649 - Supplemental material for Safety reporting quality in multiple sclerosis clinical trials: A review of phase III clinical trials included in FDA approval of disease-modifying treatments [file sj-docx-1-mso-10.1177_20552173251390649.docx]

**Supplementary Material**

**Supplemental Table 1.** Characteristics of randomized-controlled clinical trials of disease-modifying therapies for the treatment of multiple sclerosis excluded due to not being part of original approval

| **Trial name(s)** | **Intervention** | **Indication** |
| --- | --- | --- |
| PARADIGMS | Fingolimod | Pediatric RRMS |
| EU-SPMS | Interferon beta-1b | SPMS |
| NA-SPMS | Interferon beta-1b | SPMS |
| BENEFIT | Interferon beta-1b | CIS |
| TOPIC | Teriflunomide | CIS |

*CIS*: clinically isolated syndrome, *RRMS*: relapsing-remitting multiple sclerosis, *SPMS*: secondary progressive multiple sclerosis.

**Supplemental Table 2.** Second set of adverse event reporting criteria (adapted from Ioannidis et al. 2004; Hadi et al. 2017).

| **Paper Section** | **CONSORT Harms extension checklist** | **Criteria List (adapted from Hadi et al., 2017)** |
| --- | --- | --- |
| Title and abstract | 1. If the study collected data on harms and benefits, the title or abstract should so state | 1. Title or abstract states if adverse events are addressed in the article |
| Introduction | 2. If the trial addresses both harms and benefits, the introduction should so state | 2. Introduction or purpose states if adverse events are addressed in the article |
| Methods | 3. List addressed adverse events with definitions for each (with attention, when relevant, to grading, expected vs. unexpected events, reference to standardized and validated definitions, and description of new definitions) | 3. Article specifies use of validated instruments for adverse event grading |
|  |  |  |
|  |  | 4. Article specifies definitions of adverse events |
|  | 4. Clarify how harms-related information was collected (mode of data collection, timing, attribution methods, intensity of ascertainment, and harms-related monitoring and stopping rules, if pertinent) | 5. Article specifies how harms data was collected |
|  |  | 6. Article specifies when harms data was collected |
|  |  | 7. Article specifies if adverse events were attributed to trial drug |
|  | 5. Describe plans for presenting and analyzing information on harms (including coding, handling of recurrent events, specification of timing issues, handling of continuous measures, and any statistical analyses) | 8. Article specifies methods for presenting and analyzing adverse events |
| Results | 6. Describe for each arm the participant withdrawals that are due to harms and their experiences with the allocated treatment | 9. Article reports number of withdrawals caused by adverse events in each arm |
|  |  | 10. Article describes adverse events leading to withdrawals |
|  |  | 11. Article describes adverse events leading to deaths |
|  | 7. Provide the denominators for analyses on harms | 12. Article reports denominators for adverse events |
|  |  | 13. Article reports definitions of population analyzed |
|  | 8. Present the absolute risk per arm and per adverse event type, grade, and seriousness, and present appropriate metrics for recurrent events, continuous variables, and scale variables, whenever pertinent | 14. Article reports results separately for each arm |
|  |  | 15. Article reports severe adverse events separately |
|  |  | 16. Article reports absolute number of adverse events and number of patients with adverse events |
|  | 9. Describe any subgroup analyses and exploratory analyses for harms | Not included: only valid in publications including subgroup analyses |
| Discussion | 10. Provide a balanced discussion of benefits and harms with emphasis on study limitations, generalizability, and other sources of information on harms | 17. Article has balanced discussion of efficacy and adverse events |
|  |  | 18. Article discusses limitations in regards to adverse events |

**Supplemental Table 3.** Number of publications (n = 30) fulfilling each criterion in the set of criteria adapted from Sivendran et al., 2014, subdivided into small molecule drugs (n = 15) and biologic drugs (n = 15), further subdivided by date of publication (2010 and prior, and after 2010, i.e., year of publication of harms extension), including supplemental data.

| **Criterion** | **Number of publications fulfilling criterion**  **n (%)** | | | | | | |
| --- | --- | --- | --- | --- | --- | --- | --- |
|  | **Small molecule drugs**  **(n = 15)** | | | **Biologic drugs**  **(n = 15)** | | | **Total**  **(n = 30)** |
|  | **2010 and prior**  **(n = 5)** | **After 2010 (n = 10)** | **Total (n = 15)** | **2010 and prior**  **(n = 7)** | **After 2010**  **(n = 8)** | **Total**  **(n = 15)** |  |
| 1. Title or abstract states if adverse events are addressed | 5 (100%) | 10 (100%) | 15 (100%) | 4 (57%) | 8 (100%) | 12 (80%) | 27 (90%) |
| 2. Introduction or purpose states if adverse events are addressed | 2 (40%) | 10 (100%) | 12 (80%) | 3 (43%) | 5 (63%) | 8 (53%) | 20 (67%) |
| 3. Article specifies if reported adverse events include all recorded events or selected sample | 4 (80%) | 10 (100%) | 14 (93%) | 2 (29%) | 8 (100%) | 10 (67%) | 24 (80%) |
| 4. Article specifies instrument or scale used to categorize lab-defined toxicities | 2 (40%) | 9 (90%) | 11 (73%) | 2 (29%) | 5 (63%) | 7 (47%) | 18 (60%) |
| 5. Article specifies instrument or scale used to categorize all other adverse events | 1 (20%) | 7 (70%) | 8 (53%) | 0 (0%) | 5 (63%) | 5 (33%) | 13 (43%) |
| 6. Article specifies surveillance time frame for adverse events | 5 (100%) | 8 (80%) | 13 (87%) | 6 (86%) | 7 (88%) | 13 (87%) | 26 (87%) |
| 7. Article specifies if early stopping rule was used | 0 (0%) | 1 (10%) | 1 (7%) | 1 (14%) | 0 (0%) | 1 (7%) | 2 (7%) |
| 8. Article specifies if recurrent events in the same patient are counted as single or separate events | 4 (80%) | 9 (90%) | 13 (87%) | 4 (57%) | 7 (88%) | 11 (73%) | 24 (80%) |
| 9. Article reports reasons for treatment discontinuations | 5 (100%) | 9 (90%) | 14 (93%) | 7 (100%) | 8 (100%) | 15 (100%) | 29 (97%) |
| 10. Article reports if deaths related to adverse events have occurred | 4 (80%) | 10 (100%) | 14 (93%) | 6 (86%) | 8 (100%) | 14 (93%) | 28 (93%) |
| 11. Article specifies which patients were evaluable for toxicity | 5 (100%) | 9 (90%) | 14 (93%) | 4 (57%) | 6 (75%) | 10 (67%) | 24 (80%) |
| 12. Article reports absolute numbers of adverse events | 5 (100%) | 10 (100%) | 15 (100%) | 1 (14%) | 8 (100%) | 9 (60%) | 24 (80%) |
| 13. Article reports all adverse events and not only those above a threshold | 3 (60%) | 10 (100%) | 13 (87%) | 1 (14%) | 7 (88%) | 8 (53%) | 21 (70%) |
| 14. Article does not combine adverse events of varying severity | 3 (60%) | 10 (100%) | 13 (87%) | 5 (71%) | 8 (100%) | 13 (87%) | 26 (87%) |
| 15. Article does not use vague descriptors of toxicity | 3 (60%) | 7 (70%) | 10 (67%) | 1 (14%) | 2 (25%) | 3 (20%) | 13 (43%) |

**Supplemental Table 4.** Coefficients table from multiple linear regression of independent variables on safety reporting scores using the first set of criteria, including supplemental data. * : *p* < 0.05, **: *p* < 0.01, ***: *p* < 0.001

| **Variable** | **β [95% confidence interval]** | ***t*-value** | ***p* value** |
| --- | --- | --- | --- |
| Year of publication | 0.623[0.302, 0.944] | 4.061 | 0.001 *** |
| Journal impact factor | 0.129[-0.131, 0.389] | 1.038 | 0.312 |
| Randomized controlled trial | 0.074[-0.196, 0.343] | 0.570 | 0.575 |
| Double-blinded | -0.175[-0.485, 0.135] | -1.183 | 0.251 |
| Location (single country) | 0.049[-0.331, 0.429] | 0.268 | 0.792 |
| Funding (industry/government) | -0.053[-0.495, 0.390] | -0.249 | 0.806 |
| Type of intervention (small molecule drug) | 0.355[0.128, 0.582] | 3.272 | 0.004 ** |
| Comparator group (placebo or no control) | 0.047[-0.210, 0.305] | 0.386 | 0.704 |
| Previous approval of active ingredient for other indication | -0.010[-0.277, 0.257] | -0.081 | 0.936 |
| Number of participants | 0.227[-0.102, 0.557] | 1.446 | 0.165 |

**Supplemental Table 5.** Number of publications (n = 30) fulfilling each criterion in the set of criteria adapted from Hadi et al., 2017, subdivided into small molecule drugs (n = 15) and biologic drugs (n = 15), further subdivided by date of publication (2010 and prior, and after 2010, i.e., year of publication of harms extension).

| **Criterion** | **Number of publications fulfilling criterion**  **n (%)** | | | | | | |
| --- | --- | --- | --- | --- | --- | --- | --- |
|  | **Small molecule drugs**  **(n = 15)** | | | **Biologic drugs**  **(n = 15)** | | | **Total**  **(n = 30)** |
|  | **2010 and prior**  **(n = 5)** | **After 2010 (n = 10)** | **Total (n = 15)** | **2010 and prior**  **(n = 7)** | **After 2010**  **(n = 8)** | **Total**  **(n = 15)** |  |
| 1. Title or abstract states if adverse events are addressed in the article | 5 (100%) | 10 (100%) | 15 (100%) | 4 (57%) | 8 (100%) | 12 (80%) | 27 (90%) |
| 2. Introduction or purpose states if adverse events are addressed in the article | 2 (40%) | 10 (100%) | 12 (80%) | 3 (43%) | 5 (63%) | 8 (53%) | 20 (67%) |
| 3. Article specifies use of validated instruments for adverse event grading | 1 (20%) | 7 (70%) | 8 (53%) | 0 (0%) | 5 (63%) | 5 (33%) | 13 (43%) |
| 4. Article specifies definitions of adverse events | 1 (20%) | 6 (60%) | 7 (47%) | 0 (0%) | 5 (63%) | 5 (33%) | 12 (40%) |
| 5. Article specifies how harms data was collected | 4 (80%) | 7 (70%) | 11 (73%) | 3 (43%) | 4 (50%) | 7 (47%) | 18 (60%) |
| 6. Article specifies when harms data was collected | 5 (100%) | 7 (70%) | 12 (80%) | 6 (86%) | 7 (88%) | 13 (87%) | 25 (83%) |
| 7. Article specifies if adverse events were attributed to trial drug | 5 (100%) | 10 (100%) | 15 (100%) | 4 (57%) | 8 (100%) | 12 (80%) | 27 (90%) |
| 8. Article specifies methods for presenting and analyzing adverse events | 1 (20%) | 5 (50%) | 6 (40%) | 3 (43%) | 4 (50%) | 7 (47%) | 13 (43%) |
| 9. Article reports number of withdrawals caused by adverse events in each arm | 5 (100%) | 9 (90%) | 14 (93%) | 7 (100%) | 8 (100%) | 15 (100%) | 29 (97%) |
| 10. Article describes adverse events leading to withdrawals | 5 (100%) | 9 (90%) | 14 (93%) | 7 (100%) | 5 (63%) | 12 (80%) | 26 (87%) |
| 11. Article describes adverse events leading to deaths | 4 (80%) | 10 (100%) | 14 (93%) | 6 (86%) | 8 (100%) | 14 (93%) | 28 (93%) |
| 12. Article reports denominators for adverse events | 4 (80%) | 10 (100%) | 14 (93%) | 5 (71%) | 8 (100%) | 13 (87%) | 27 (90%) |
| 13. Article reports definitions of population analyzed | 5 (100%) | 10 (100%) | 15 (100%) | 4 (57%) | 7 (88%) | 11 (73%) | 26 (87%) |
| 14. Article reports results separately for each arm | 5 (100%) | 9 (90%) | 14 (93%) | 7 (100%) | 8 (100%) | 15 (100%) | 29 (27%) |
| 15. Article reports severe adverse events separately | 3 (60%) | 10 (100%) | 13 (87%) | 4 (57%) | 8 (100%) | 12 (80%) | 25 (83%) |
| 16. Article reports absolute number of adverse events and number of patients with adverse events | 5 (100%) | 10 (100%) | 15 (100%) | 1 (14%) | 8 (100%) | 9 (60%) | 24 (80%) |
| 17. Article has balanced discussion of efficacy and adverse events | 4 (80%) | 5 (50%) | 9 (60%) | 3 (43%) | 6 (75%) | 9 (60%) | 18 (60%) |
| 18. Article discusses limitations in regards to adverse events | 2 (40%) | 4 (40%) | 6 (40%) | 1 (14%) | 7 (88%) | 8 (53%) | 14 (47%) |

**Supplemental Table 6.** Coefficients table from multiple linear regression of independent variables on safety reporting scores from Hadi et al. * : *p* < 0.05, **: *p* < 0.01

| **Variable** | **β [95% confidence interval]** | ***t*-value** | ***p* value** |
| --- | --- | --- | --- |
| Year of publication | 0.749 [0.286, 1.211] | 3.388 | 0.003 ** |
| Journal impact factor | 0.111 [-0.264, 0.486] | 0.619 | 0.543 |
| Randomized controlled trial | -0.021 [-0.410, 0.368] | -0.112 | 0.912 |
| Double-blinded | -0.107 [-0.553, 0.340] | -0.500 | 0.623 |
| Location (single country) | -0.079 [-0.626, 0.469] | -0.301 | 0.767 |
| Funding (industry/government) | 0.106 [-0.532, 0.744] | 0.347 | 0.732 |
| Type of intervention (small molecule drug) | 0.125 [-0.202, 0.453] | 0.802 | 0.433 |
| Comparator group (placebo or single-arm) | 0.036 [-0.335, 0.406] | 0.201 | 0.842 |
| Previous approval of active ingredient for other indication | 0.120 [-0.265, 0.504] | 0.653 | 0.522 |
| Number of participants | 0.018 [-0.456, 0.493] | 0.081 | 0.936 |
